# Regenerative potential of nanoenabled collagen-polylactide scaffolds for osteochondral defect repair in rabbits

**DOI:** 10.3389/fbioe.2025.1699338

**Published:** 2025-12-01

**Authors:** Lena Schröter, Graciosa Quelhas Teixeira, Luisa de Roy, Benjamin Thilo Krüger, Oliver Küppers, Catarina Leite Pereira, Daniela Pereira Vasconcelos, Meriem Lamghari, Bruno Sarmento, Geir Klinkenberg, Ruth Schmid, Raquel Gracia, Janire Alkorta, Damien Dupin, Anne-Marie Haaparanta, Virpi Muhonen, Andreas Martin Seitz, Anita Ignatius

**Affiliations:** 1 Institute of Orthopaedic Research and Biomechanics, Ulm University Medical Center, Ulm, Germany; 2 Instituto de Investigação e Inovação em Saúde (i3S), Instituto Nacional de Engenharia Biomédica (INEB), Universidade do Porto, Porto, Portugal; 3 SINTEF Industry, Department of Biotechnology and Nanomedicine, Trondheim, Norway; 4 CIDETEC, Basque Research and Technology Alliance (BRTA), Parque Científico y Tecnológico de Gipuzkoa, Donostia-San Sebastián, Spain; 5 Askel Healthcare Ltd., Tampere, Finland

**Keywords:** biomaterials, cartilage tissue engineering, nanoenabled scaffolds, inflammation, immunomodulation, osteoarthritis

## Abstract

Current tissue engineering strategies for treating traumatic or degenerative cartilage defects in osteoarthritis (OA) remain insufficient in promoting robust tissue regeneration while simultaneously addressing inflammation, matrix degradation, and post-surgical infections. In this study, we evaluated the *in vivo* biocompatibility and regenerative potential of a nanoenabled collagen-polylactide (Col-PLA) scaffold functionalized with tri-combinatorial nanoemulsions delivering ibuprofen (anti-inflammatory), batimastat (BB-94, anti-proteolytic), and mupirocin (anti-bacterial). Using a femoral osteochondral defect model in New Zealand White rabbits, regeneration was assessed at 4- and 12-week post-implantation by macroscopic scoring, biomechanical indentation mapping, and histological analysis. Synovial inflammation was further evaluated via histology, CD8 immunostaining, and quantification of key pro-inflammatory mediators including interleukin-1β (IL-1β), tumor necrosis factor-α (TNF-α), prostaglandin E_2_ (PGE_2_), and matrix metalloproteinase-13 (MMP-13). Both functionalized and non-functionalized Col-PLA scaffolds supported significant host cell infiltration and tissue regeneration, outperforming untreated controls and demonstrating effective subchondral bone repair. A transient inflammatory response was observed in the nanoenabled group at 4 weeks, without elevation of synovial pro-inflammatory cytokines or compromised tissue regeneration. Although cartilage repair was comparable between scaffold groups, the nanoenabled Col-PLA scaffold might have a potential benefit in more complex or comorbid clinical scenarios due to its immunomodulatory, anti-proteolytic, and anti-microbial functionalization. The findings of this study support further investigations of these modular scaffolds in OA and infection-prone environments, using disease-relevant and long-term models, to fully establish its therapeutic applicability in regenerative medicine.

## 1 Introduction

Articular cartilage injuries are common and remain a major challenge in regenerative medicine due to the tissue’s limited self-healing capacity (Wang M et al., 2024). Current clinical treatments, such as microfracture (Steadman et al., 2001) and autologous chondrocyte implantation (ACI) (Brittberg et al., 1994), often lead to the formation of fibrocartilage, resulting in suboptimal long-term outcomes (Falah et al., 2010; Medvedeva et al., 2018). Incomplete repair increases the risk of developing osteoarthritis (OA), a progressive, multifactorial joint disease characterized by cartilage degradation, chronic inflammation, and joint dysfunction (Goldring and Otero, 2011; de Lange-Brokaar et al., 2012; Wojdasiewicz et al., 2014; Mathiessen and Conaghan, 2017), which ultimately leads to severe joint pain and failure (Steinmetz et al., 2023). As OA continues to rise globally, its socioeconomic impact grows accordingly (Steinmetz et al., 2023).

Tissue engineering offers promising alternatives, particularly using three-dimensional (3D) scaffolds that mimic the extracellular matrix (ECM) to support cell adhesion, migration, proliferation, and differentiation (Chen and Kawazoe, 2018). Ideal scaffolds should be biocompatible, biodegradable at a rate matching tissue regeneration, and mechanically stable. Synthetic polymers such as polylactide (PLA) and poly(lactide-co-glycolide) (PLGA), as well as natural materials like chitosan and collagen, have been widely used in scaffold design for cartilage repair (Medvedeva et al., 2018). These materials have also been combined to create composite scaffolds that merge the favorable mechanical properties of synthetic polymers with the biocompatibility and bioactivity of natural components. A promising approach has been the development of a hybrid scaffold based on collagen (Col) and PLA (Col-PLA), combining the hydrophilic collagen component with the biomechanical stability of the synthetic polymer. The scaffold displayed good mechanical and cell-attachment properties (Haaparanta et al., 2014) and the treatment of cartilage defects in an *in vivo* porcine model resulted in improved tissue repair (Muhonen et al., 2016).

Building on these developments, recent research has increasingly focused on integrating nanotechnology into scaffold systems to enable localized, controlled drug delivery and to enhance regenerative outcomes (Eftekhari et al., 2020; Leite Pereira et al., 2022; Qiao et al., 2022; Pereira Vasconcelos et al., 2024). Among these, nanoemulsions have emerged as particularly promising carriers due to their ability to encapsulate both hydrophilic and hydrophobic therapeutic agents (Navascuez et al., 2021; Wilson et al., 2022; Kumar et al., 2024). This multi-drug delivery strategy allows for the targeted modulation of key pathological processes at the injury site, including inflammation, proteolysis, and infection, while minimizing systemic side effects often associated with conventional treatments (Eftekhari et al., 2020; Menon et al., 2023; Kumar et al., 2024). Ibuprofen, a non-steroidal anti-inflammatory drug (NSAID), can suppress local inflammation via cyclooxygenase (COX) inhibition, thereby inhibiting COX-mediated prostaglandin (PG) production (Nakata et al., 2018). Batimastat (BB-94), a broad-spectrum matrix metalloproteinase (MMP) inhibitor, may prevent cartilage breakdown and OA progression (Rengel et al., 2007). Moreover, the integration of anti-bacterial substances into 3D matrices emerged as a strategy to prevent the potential infection risk associated with surgical procedures such as scaffold implantation (Leite Pereira et al., 2022).

In a previous study (Pereira Vasconcelos et al., 2024), a novel, biodegradable and nanoenabled scaffold was successfully produced by the conjugation of a collagen-polylactide (Col-PLA) scaffold based on Haaparanta et al. (2014) with PLGA-based ibuprofen-loaded nanocarriers, displaying immunomodulatory capacities by reducing immune cell recruitment both *in vitro* and *in vivo* in a rodent air-pouch model. Taking this into consideration, the main aim of this work is to develop a nanoenabled Col-PLA scaffold. Nanoemulsions stabilized by dextran-based single-chain polymer nanoparticles (DXT SCPNs) and co-loaded with ibuprofen, BB-94, and mupirocin are incorporated into the Col-PLA matrix to provide anti-inflammatory, anti-bacterial, and anti-proteolytic properties. The potential of this novel nanoenabled Col-PLA scaffold to support cartilage repair is investigated by evaluating its regenerative capacity, biocompatibility, and immunomodulatory effects after 4 and 12 weeks of implantation in osteochondral defects in New Zealand White rabbits.

## 2 Materials and methods

### 2.1 Production of oil-in-water (O/W) emulsions

Dextran single-chain polymer nanoparticles were prepared from methacrylate-functionalized dextran (DXT-MA), a polysaccharide with a degree of substitution of methacrylate group (DS_DXT-MA_; percentage of modified hydroxyl groups per repeating unit) of 40%, as previously described (Gracia et al., 2017). Additionally, an intra-crosslinking reaction was employed to obtain dextran-based single-chain polymer nanoparticles (DXT-SCPN-MA, Supplementary Figure S1) with a final degree of substitution of methacrylate group (DS_SCPN-MA_) of 20% (Supplementary Figure S2). Detailed descriptions of these methods are provided in the Supplementary Material and Methods. In an 8 mL glass vial, DXT-SCPN-MA were redispersed in 4.5 mL of deionized water at 0.11 mg/mL, followed by the dissolution of Ibuprofen salt at 30 mg/mL concentration. Separately, BB-94 and mupirocin were dissolved in sunflower oil to reach 0.19 and 6 mg/mL. Afterwards, 0.5 g of this oil solution was added to the redispersed DXT-SCPN-MA aqueous solution, resulting in a biphasic system due to the immiscibility of both phases. To obtain a tricombinatorial emulsion with 0.019 mg/mL BB-94, 0.6 mg/mL mupirocin and 30 mg/mL Ibuprofen, both phases were sonicated (0 °C, no stirring) using an UP400S (Hielscher) system at 100% of amplitude and pulse for 4 min (400 W) with a H3 sonotrode tip (3 mm diameter, 100 mm length), as previously described (Navascuez et al., 2021). Dynamic light scattering (DLS) analysis was performed using a Zetasizer Nano ZS, ZEN3600 Model (Malvern Instruments Ltd) to determine the oil droplet size (Z-average: 195 nm, PDI: 0.2, see Supplementary Figure S3). All measurements were performed in disposable sizing cuvettes at a laser wavelength of 633 nm and a scattering angle of 173°. Quantification of the formulated drugs was determined by High Performance Liquid Chromatography (HPLC). The HPLC set-up for each compound is described in the Supplementary Material.

### 2.2 Col-PLA scaffold and nanoenabled Col-PLA scaffold preparation

Col-PLA scaffolds were prepared as previously described (Pereira Vasconcelos et al., 2024). Briefly, bovine type I collagen (PureCol, Advanced Biomatrix, Carlsbad, US) was added to the PLA (Askel Healthcare, Helsinki, Finland) mesh prior to freeze-drying. Freeze-dried scaffolds were subsequently cross-linked with N(3-dimethylaminopropyl)-N′-ethylcarbodiimide hydrochloride (EDC) and N-hydroxysuccinimide (NHS) (Merck Life Science S.L.U., Algés, Portugal). To form the nanoenabled Col-PLA scaffolds, tri-combinatorial nanoemulsions were incorporated through a physical mixture of the nanoemulsions with the collagenous component of the scaffold to achieve a final concentration of 3 mg/mL ibuprofen, 0.0019 mg/mL BB-94 and 0.06 mg/mL mupirocin per scaffold. Scaffolds were freeze-dried and crosslinked by NHS-EDC chemistry. All scaffolds were prepared under sterile conditions.

### 2.3 Safety assessment of the Col-PLA scaffolds

#### 2.3.1 Evaluation of endotoxin content and bacterial contamination

Sterility and endotoxin determination are part of the safety assessment of implantable medical devices. It is crucial to ensure a sterile production of the Col-PLA scaffolds, as bacterial or endotoxin contamination may influence the *in vitro* and *in vivo* results and lead to false conclusions about the safety and efficacy of novel implants. Samples of empty and nanoenabled Col-PLA scaffolds were extracted overnight at room temperature in endotoxin free water according to FDA guidelines (U.S. Food and Drug Administration, 2012). The FDA guidelines recommend that 10 devices should be extracted in 40 mL water. However, due to the small size and the limited supply of the sample scaffolds, three parallel extractions of sample scaffolds in 1 mL of endotoxin water were applied for each sample type. The samples were tested for endotoxin content with the PyroGene Recombinant Factor C kit (Lonza) according to the protocol described in the kit. All the equipment applied was classified as endotoxin free. The sample extracts and samples were tested for bacterial contamination by plating on 3M Petrifilm Aerobic Count Plates. The samples were diluted 10-, 100-, 1000-, 10000- and 100000-fold in sterile PBSW (Oxoid, BR0014) and a volume of 1 mL was subjected onto the Petrifilms. Samples were incubated at 35 °C for 72 h before the number of visible colonies on the Petrifilms were counted.

#### 2.3.2 Evaluation of *in vitro* cytotoxicity

All Col-PLA scaffolds were extracted in cell culture medium and tested for cytotoxicity according to ISO 10993-12 and ISO-10993-5 guidelines. The scaffolds were extracted in cell culture medium for 24 h at 37 °C and tested for cytotoxicity according to validated SOPs from the European Nanomedicine Characterization Laboratory (EUNCL-GTA01, EUNCL-GTA02 and EUNCL-GTA03 with LLC-PK1, Hep G2 and L929 cell lines).

### 2.4 Animal study

The animal study was approved by the Local Ethical Committee (*Regierungspräsidium Tübingen*, Germany; Approval No. 1479) and conducted in compliance with the European Union Directive 2010/63/EU on the protection of animals used for scientific purposes. A total of 27 adult female New Zealand White rabbits (age: 24 weeks; mean weight: 3.5 kg) were included in the study. Of these, 24 animals underwent bilateral surgery in which an osteochondral defect was created in the medial femoral condyle of each knee. The defects were treated with either a Col-PLA scaffold (n = 8), a nanoenabled Col-PLA scaffold (n = 8), or left untreated as an empty control (n = 8). The remaining three animals did not undergo surgery; the intact cartilage in both the left and right medial femoral condyles of these rabbits was used as a physiological control.

### 2.5 Surgical procedure

All surgical procedures were performed under general anesthesia, induced and maintained via intravenous injection of ketamine hydrochloride (7.5–15 mg/kg body weight [BW]) combined with xylazine hydrochloride (0.5–1 mg/kg BW). A medial parapatellar arthrotomy with lateral patellar dislocation was used to access the medial femoral condyle. An osteochondral defect (3 mm diameter, 2 mm depth) was created in the load-bearing region using surgical drills and a milling cutter. The defect size and location were selected based on the *American Society for Testing and Materials* guidelines, which state that full-thickness cartilage defects larger than 3 mm do not spontaneously regenerate without intervention (ASTM International, 2010). Bone and cartilage debris were removed by saline irrigation, and the defect was dried using a swab. Cylindrical scaffolds (3 mm diameter) were prepared from Col-PLA or nanoenabled Col-PLA scaffold sheets using a biopsy punch to match the defect dimensions. The scaffolds were then inserted into the defects and secured with fibrin glue (TISSEEL^®^, Baxter GmbH, Unterschleißheim, Germany) applied around the scaffold borders. In the control group, defects were left empty. Following implantation, the joint capsule, subcutaneous tissue, and skin were closed in layers with resorbable sutures. Perioperative and postoperative analgesia included tramadol (25 mg/mL) administered via drinking water for 3 days before and after surgery, along with a preoperative subcutaneous injection of buprenorphine (0.03 mg/kg BW). To assess early and late cellular responses, animals were sacrificed at either 4- or 12-week post-surgery. Rabbits were humanely euthanized by mechanical stunning with a captive bolt gun, immediately followed by exsanguination via major vessel transection. This procedure ensured rapid loss of consciousness, minimized nociception, and produced death through hypovolemic shock. These time points were selected to capture distinct phases of osteochondral healing: week 4 represents the early inflammatory and cell infiltration stage, while week 12 reflects mid-term tissue remodeling and scaffold integration (Hunziker, 2002; Mainil-Varlet et al., 2003; Wang et al., 2015). The distal femur, synovial membrane, and synovial fluid were then harvested for further analysis.

### 2.6 Macroscopic scoring

The regenerative tissue within the defect in the medial femoral condyle was macroscopically assessed using the *International Cartilage Repair Society* (ICRS) cartilage repair assessment score as shown in Table 1. On the basis of the degree of defect repair, degree of integration and macroscopic appearance, the cartilage regeneration was rated in four different grades from grade I (normal) to grade IV (severely abnormal) (Brittberg and Peterson, 1998; Peterson et al., 2000).

**TABLE 1:** International Cartilage Repair Society (ICRS) cartilage repair assessment score (Brittberg and Peterson, 1998; Peterson et al., 2000).

	Criteria	Points
Degree of defect repair	Level with surrounding cartilage 75% repair of defect depth 50% repair of defect depth 25% repair of defect depth 0% repair of defect depth	4 3 2 1 0
Integration to border zone	Complete integration with surrounding cartilage Demarcating border <1 mm 3/4th of graft integrated, 1/4th with a notable border >1 mm width 1/2 of graft integrated with surrounding cartilage, 1/2 with a notable border >1 mm From no contact to 1/4th of graft integrated with surrounding cartilage	4 3 2 1 0
Macroscopic appearance	Intact smooth surface Fibrillated surface Small, scattered fissures or cracks Several small or few but large fissures Total degeneration of grafted area	4 3 2 1 0
Overall repair assessment	Grade I: normal Grade II: nearly normal Grade III: abnormal Grade IV: severely abnormal	12 11–8 7–4 3–1

### 2.7 Biomechanical indentation mapping

**FIGURE 1:**
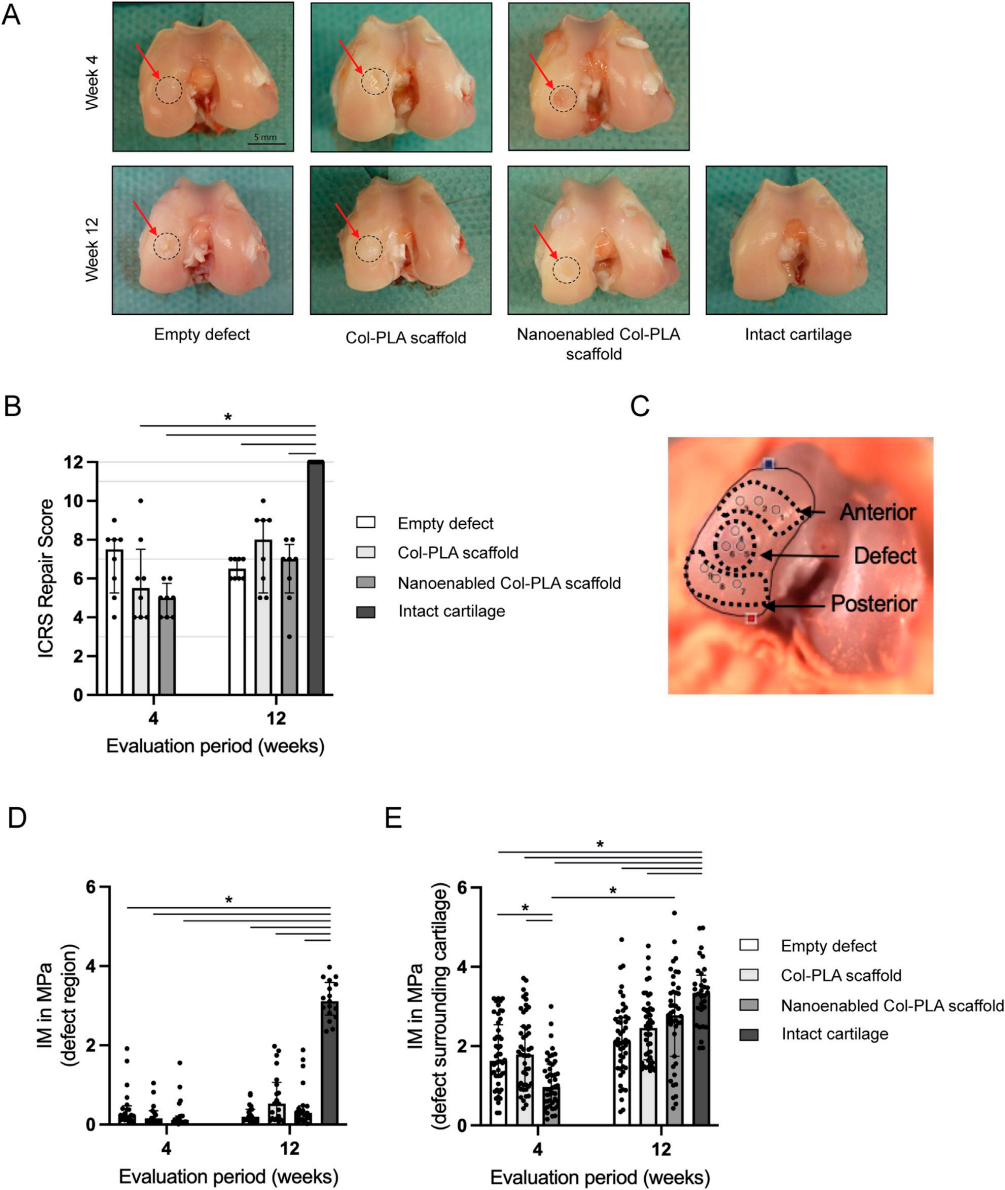
Macroscopic assessment of cartilage repair and biomechanical evaluation of initial elastic response. **(A)** Representative macroscopic images of osteochondral defects in the empty defect group, Col-PLA scaffold group, and nanoenabled Col-PLA scaffold group at 4- and 12-week post-surgery, alongside the intact (non-operated) cartilage control. Cartilage-like regenerative tissue is visible within the defect sites (red arrows). Scale bar: 5 mm. **(B)** Semi-quantitative macroscopic evaluation of cartilage repair using the ICRS scoring system. All surgical groups showed significantly lower scores compared to the intact cartilage group, except for the Col-PLA scaffold group at 12 weeks. **(C)** Schematic representation of measurement locations in the defect center and surrounding cartilage (anterior and posterior) using a spherical indenter. **(D)** Instantaneous modulus (IM, MPa) in the defect region and **(E)** in the surrounding cartilage of the empty defect, Col-PLA scaffold, and nanoenabled Col-PLA scaffold groups at 4 and 12 weeks, compared to intact cartilage. Data are presented as median ± interquartile range (n = 6–8). Statistical analysis: Kruskal–Wallis test followed by Dunn’s multiple comparisons test; *p ≤ 0.05.

Following macroscopic scoring, biomechanical indentation mapping (Seitz et al., 2021) of the medial femoral condyle was performed to detect biomechanical changes in the defect region and the surrounding cartilage after implantation. Briefly, a multiaxial mechanical tester (MACH-1 v500css, Biomomentum Inc., Laval, QC, Canada) equipped with a 17 N load cell was used to perform spatial normal indentation tests on the surface of the medial femur in accordance to an established algorithm (Sim et al., 2017a). Depending on the animal group, the defect area (with or without the implanted scaffolds) and the defect-surrounding cartilage or the intact cartilage were tested. First, the built-in camera-registration system was used to define a pattern with measurement points on the surface of the defect (n = 3 measurement points) and its surrounding cartilage (n = 6 measurement points) (Figure 1A). On each measurement point a non-destructive indentation relaxation test was performed using a spherical indenter (Ø = 1 mm, indentation depth: 0.06 mm, velocity: 0.1 mm/s, relaxation time: 10 s). Subsequently, the cartilage or the repair tissue thickness was evaluated by needle penetration tests (Sim et al., 2017b). The experimental data were analyzed on the basis of a mathematical least squares fitting of an elastic model to determine the instantaneous modulus (IM, in MPa) as a measure of the initial material elasticity (Hayes et al., 1972).

### 2.8 Histology

For histological analysis of the medial femoral condyle, the formalin-fixed distal femur was bisected in the longitudinal axis, and the medial femoral condyles were decalcified using 20% ethylenediaminetetraacetic acid (pH 7.2–7.4) for >35 days. Subsequently, the samples were dehydrated in an ascending ethanol series, embedded in paraffin and cut into sections of 4 μm using a rotary microtome. For histological analyses, sections were stained with safranin-O/fast green for an overall assessment of the tissue and to detect proteoglycans. The sections were examined by standard bright-field light microscopy (Leica DMI6000 B, Leica, Heerbrugg, Switzerland). The quality of the repair tissue was evaluated using the O’Driscoll Scoring System as shown in Table 2 (O'Driscoll et al., 1986; O'Driscoll, 1998).

**TABLE 2:** O’Driscoll Scoring System for cartilage repair assessment score (O'Driscoll et al., 1986; O'Driscoll, 1998).

Histological parameter		Score
Nature of predominant tissue	Cellular morphology: Hyaline articular cartilage Incompletely differentiated mesenchyme Fibrous tissue/bone	4 2 0
	Safranin-O staining of the matrix: Normal or nearly normal Moderate Slight None	3 2 1 0
Structural characteristics	Surface regularity: Smooth and intact Superficial horizontal lamination Fissures: 25%–100% of the thickness Severe disruption, including fibrillation	3 2 1 0
	Structural integrity: Normal Slight disruption, including cysts Severe disintegration	2 1 0
	Thickness: 100% of normal adjacent cartilage 50%–100% of normal cartilage <50% of normal cartilage	2 1 0
	Bonding to adjacent cartilage: Bonded at both ends of graft Bonded at one end, or partially at both ends Not bonded	2 1 0
Absence of cellular changes resulting from degeneration	Hypocellularity: Normal cellularity Slight hypocellularity Moderate hypocellularity Severe hypocellularity	3 2 1 0
	Chondrocyte clustering: No clusters <25% of the cells 25%–100% of the cells	2 1 0
	Absence of degenerative changes in adjacent cartilage: Normal cellularity, no clusters, normal staining Normal cellularity, mild clusters, moderate staining Mild or moderate hypocellularity, slight staining Severe hypocellularity, poor or no staining	3 2 1 0
Total		24

Picrosirius red staining (Abcam, Cambridge, UK) was performed to evaluate the ultrastructure of the regenerative tissue under polarized light, which is mainly reflected by the structure and orientation of collagen fibers. Birefringent collagen fibers were imaged with polarized light (Axiophot 451887, Zeiss, Jena, Germany). The color hue corresponds to both fiber thickness and packing of the fibers: green to greenish yellow represents thin or loosely packed collagen fibers, whereas yellowish orange through orange to red indicates thick or tightly packed collagen fibers (Montes and Junqueira, 1991). Moreover, collagen type I may present as thick (2–10 μm in diameter), closely packed and strongly birefringent, yellow or red fibers, whereas type II collagen forms loosely packed fibrils (20–30 nm in diameter) with a weak birefringence of a varying color. All images were captured with the same parameters. Area of red (1–9 nm; 230–255 nm), orange (10–38 nm), yellow (39–51 nm) and green (52–128 nm) fibers were quantified using ImageJ software following Widmayer et al. (2023).

The synovial membrane of the knee joint was fixed in 4% buffered formalin solution before it was dehydrated in an ascending ethanol series, embedded in paraffin and cut into 4 μm sections. For histological evaluation, the sections of the synovial membrane were stained with hematoxylin and eosin and analyzed using an OA grading system (Table 3).

**TABLE 3:** Histopathologic assessment of osteoarthritis synoviopathy (Krenn et al., 2002).

Histological parameter		Points
Enlargement of the synovial lining cell layer	The lining cells form one layer The lining cells form 2–3 layers The lining cells form 4–5 layers, few multinucleated cells might occur The lining cells form more than 5 layers, the lining might be ulcerated and multinucleated cells might occur	0 1 2 3
Density of the resident cells	The synovial stroma shows normal cellularity The cellularity is slightly increased The cellularity is moderately increased, multinucleated cells might occur The cellularity is greatly increased, multinucleated giant cells, pannus formation and rheumatoid granulomas might occur	0 1 2 3
Inflammatory infiltrate	No inflammatory infiltrate Few mostly perivascular situated lymphocytes or plasma cells Numerous lymphocytes or plasma cells, sometimes forming follicle-like aggregates Dense band-like inflammatory infiltrate or numerous large follicle-like aggregates	0 1 2 3
Score	No synovitis Low-grade synovitis High-grade synovitis	Sum 0 or 1 Sum 2–4 Sum 5–9

### 2.9 Immunohistochemistry

The identification of CD8 T lymphocytes in the synovial membranes was assessed by avidin–biotin complex (Vector laboratories, Newark, CA, USA) immunohistochemistry using the NovaRED Peroxidase Substrate Kit (Vector laboratories). Antigen retrieval was performed through incubation with 10 mM citrate buffer (pH 6.0) at 95 °C for 20 min. Following cooling for 20 min, blocking was performed with 3% H_2_O_2_ in tris-buffered saline (TBS) for 15 min at room temperature and 5% bovine serum albumin (Sigma-Aldrich, St. Louis, MO, USA) in TBS with 0.1% Tween-20 for 1 h at 37 °C. Sections were incubated with monoclonal mouse anti-CD8 (1:150 dilution, NB100-54021H, clone 12.C7, Novus Biologicals, Wiesbaden-Nordenstadt, Germany) antibodies, overnight at 4 °C, followed by incubation with goat anti-mouse IgG (H + L), Biotin-XX (1:200 dilution, Invitrogen, Waltham, MA, EUA) antibody. Isotype controls were stained with mouse IgG1 antibody (BioLegend, San Diego, CA, USA). The stained sections were imaged by light microscopy (Zeiss).

### 2.10 Protein quantification

The levels of interleukin-1β (IL-1β), tumor necrosis factor-α (TNF-α), PGE_2_ and MMP-13 in the synovial fluid were determined using different enzyme-linked immunosorbent assay kits (IL-1β: LifeSpan Biosciences, Inc., Seattle, WA, USA; PGE_2_: Arbor Assays, Ann Arbor, MI, USA; TNF-α: RayBiotech, Norcross, GA, USA; MMP-13: MyBioSource, Inc., San Diego, CA, USA) according to the manufacturers’ instructions.

### 2.11 Statistical analysis

GraphPad Prism 9 software (GraphPad Software, Inc, La Jolla, CA, USA) was used for the statistical analysis. Normal distribution was assessed with the Shapiro-Wilk test. All data were non-normally distributed. Differences between the empty defect, Col-PLA scaffold, nanoenabled Col-PLA scaffold and intact cartilage groups were analyzed using the Kruskal-Wallis test combined with Dunn’s multiple comparison test. Data are presented in the figures as median ± interquartile range. The level of significance was defined as *p* ≤ 0.05.

## 3 Results

### 3.1 Characterization of the nanoemulsion and its incorporation in the Col-PLA scaffold

The nanoemulsion to be incorporated into the Col-PLA scaffold exhibited an average particle diameter of approximately 195 nm with a polydispersity index (PDI) of 0.2, indicating a uniform size distribution as determined by DLS (Supplementary Figure S3). All the oil was encapsulated as no oil droplets could be observed at the surface of the dispersion. In addition, HPLC studies confirmed that the encapsulation efficiency was approximately 100%, with concentrations of BB-94, mupirocin and ibuprofen at 0.0019 mg/mL, 0.06 mg/mL and 30 mg/mL, respectively, as well as the chemical integrity of the three drugs. In addition, drug release studies were performed by placing the nanoemulsion in dialysis tubing (Mw cut-off 3,500 g/mol) and excess water. The release was monitored by HPLC (Supplementary Figure S4). A burst release of ibuprofen was observed within 4 h, consistent with its dissolution in the aqueous phase. In contrast, the two hydrophobic drugs, BB-94 and mupirocin, exhibited more sustained release kinetics. After 9 h, complete release of BB-94 was detected, while only approximately 40% of mupirocin was released. Furthermore, to evaluate the successful incorporation of polymeric nanocarriers into the collagen scaffolds COPLA® and to assess their impact on scaffold structure, SEM analysis was performed. The results showed highly porous scaffolds with a morphology similar to that of the control (plain collagen scaffold), which showed that the incorporation of the nanoemulsion did not affect significantly the structure of the scaffold. Moreover, the presence of spherical morphologies from the nanoemulsion was observed after freeze-drying (Supplementary Figure S5, nanoemulsions indicated by the arrow at the higher magnification). In addition, safety assessments of the nanoenabled Col-PLA scaffold confirmed sterility, with microbial contamination below 10 CFU/mL in all samples, and endotoxin levels below 0.005 EU/mL. Both values were below the acceptable regulatory limits for implantable medical devices.

### 3.2 Clinical observations and macroscopic analysis

All surgical procedures were completed without intraoperative complications. Postoperatively, all animals demonstrated normal wound healing and recovered physiological gait patterns within 3 days. Prior to euthanasia, physiological flexion and extension of the hindlimbs were confirmed in all operated rabbits.

As expected, the physiological control group (no surgery) exhibited no macroscopic signs of joint inflammation or cartilage degeneration. In contrast, mild synovial membrane redness and a slight increase in synovial fluid volume were observed in the empty defect and in both Col-PLA scaffold groups at 4 and 12 weeks postoperatively. These findings, consistent across the three groups, suggest a mild residual inflammatory response to surgical trauma. In the nanoenabled Col-PLA scaffold group, 6 out of 8 rabbit knees displayed slightly increased synovial redness and fluid volume at 4 weeks, indicative of a heightened early inflammatory response. However, by 12 weeks, these parameters were comparable across all surgical groups. Macroscopically, the defect area remained noticeable in all operated joints, with cartilage-like tissue observed within the defect sites (Figure 1A).

Semi-quantitative macroscopic evaluation using the ICRS Cartilage Repair Assessment Score (Table 1) revealed no statistically significant differences among the surgical groups. The empty defect group showed limited cartilage regeneration, with median scores of 7.5/12 at 4 weeks and 6.5/12 at 12 weeks, corresponding to Grade III (abnormal) repair at the later time point (Figure 1B). The Col-PLA scaffold group exhibited a median score of 5.5/12 (Grade III) at 4 weeks, which improved to 8/12 (Grade II) at 12 weeks. Similarly, the nanoenabled Col-PLA scaffold group scored 5/12 (Grade III) at 4 weeks and 7/12 (Grade III) at 12 weeks.

### 3.3 Biomechanical indentation mapping

The biomechanical characterization of the scaffolds showed comparable compressive strength and viscoelastic behavior between Col-PLA and nanoenabled Col-PLA scaffolds, with the latter demonstrating higher permeability (Supplementary Figure S6). The IM measurements within the defect region revealed no significant differences between the empty defect, Col-PLA scaffold, and nanoenabled Col-PLA scaffold groups at either 4- or 12-week post-surgery (Figure 1D). In contrast, analysis of the cartilage adjacent to the defect area demonstrated a significant reduction in IM values in the nanoenabled Col-PLA scaffold group at 4 weeks, compared to both the empty defect group (*p* < 0.05) and the Col-PLA scaffold group (*p* < 0.01, Figure 1E). Notably, the nanoenabled Col-PLA group exhibited a significant increase in the IM of the surrounding cartilage between 4 and 12 weeks, indicating a recovery of mechanical integrity over time. By 12 weeks, the IM values in the surrounding cartilage of this group were no longer significantly different from those of the intact cartilage. No significant changes in IM were observed over time in the empty defect or Col-PLA scaffold groups, suggesting limited biomechanical improvement in the surrounding native cartilage for these treatment conditions.

### 3.4 Histological analysis

**FIGURE 2:**
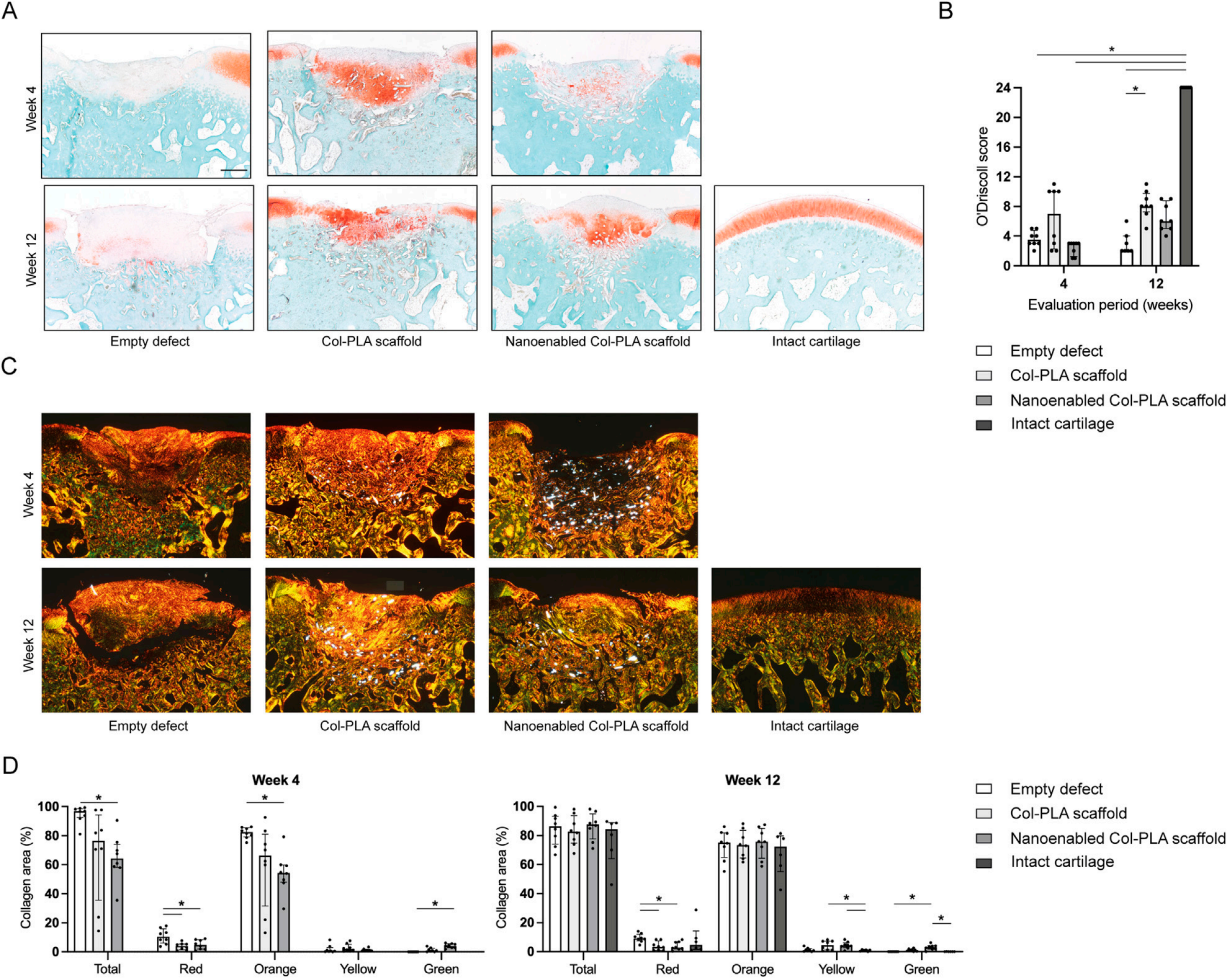
Histological evaluation of bone and cartilage tissue regeneration. **(A)** Representative safranin-O/fast green-stained sections of femoral condyles from the empty defect, Col-PLA scaffold, and nanoenabled Col-PLA scaffold groups at 4- and 12-week post-surgery, alongside the intact (non-operated) cartilage control. Safranin-O staining (red) indicates proteoglycan-rich cartilage matrix. Both scaffold-treated groups exhibited positive Safranin-O staining within the regenerative tissue, reflecting proteoglycan production. Scale bar: 500 µm. **(B)** Semi-quantitative histological evaluation of cartilage repair using the O’Driscoll scoring system. At 12 weeks, no significant differences were observed between the Col-PLA and nanoenabled Col-PLA scaffold groups; however, both appeared to show lower values compared to the intact cartilage group. The empty defect group displayed significantly lower scores compared to intact cartilage. **(C)** Representative images of picrosirius red-stained sections under polarized light for collagen fiber analysis. Green to greenish-yellow hues indicate thin or loosely packed collagen fibrils, while yellow to red hues indicate thicker or more tightly packed collagen fibers. Scale bar: 500 µm. **(D)** Quantitative analysis of collagen fiber composition at 4- and 12-week post-surgery, expressed as the percentage area of total collagen and individual fiber color types (red, orange, yellow, green). Data are presented as median ± interquartile range (n = 6–8). Statistical analysis was performed using Kruskal–Wallis test with Dunn’s multiple comparisons test; *p ≤ 0.05.

The subchondral bone beneath the defects exhibited a physiological appearance in all samples, with no evidence of osteolysis or subchondral cyst formation. These findings align with qualitative microcomputed tomography results, which demonstrated ongoing bone regeneration across all surgical groups. At both 4- and 12-week post-implantation, trabecular-like structures were observed at the margins of the osteochondral defects in the empty defect, Col-PLA scaffold, and nanoenabled Col-PLA scaffold groups (Supplementary Figure S7, Supplementary Data), indicating active bone remodeling. Histological evaluation of safranin-O-stained femoral condyle sections further confirmed these observations. In the intact cartilage group (no surgery), cartilage displayed a normal structure with uniform matrix staining and no signs of degeneration or inflammation (Figure 2A). In contrast, the empty defect group exhibited poor healing at both 4 and 12 weeks, characterized by fibrous tissue filling the defect and minimal safranin-O staining, indicative of a low proteoglycan content. Defects treated with Col-PLA scaffolds demonstrated the formation of cartilage-like tissue, with increasing proteoglycan content as reflected by safranin-O positivity. Matrix staining was observed in 4 of 8 samples at 4 weeks, increasing to 6 of 8 samples by 12 weeks. The Col-PLA scaffolds appeared well integrated with surrounding native cartilage at both time points, suggesting successful host cell infiltration and tissue remodeling. At 4 weeks, regenerative tissue in the nanoenabled Col-PLA scaffold group displayed lower structural quality compared to the Col-PLA group, with reduced surface regularity, matrix thickness, structural integrity, and bonding to adjacent cartilage (Figure 2A). Additionally, proteoglycan content was visibly lower at this time point. However, tissue structure markedly improved by 12 weeks, with 7 of 8 samples demonstrating safranin-O-positive matrix, indicating increased proteoglycan deposition. Scaffold integration with surrounding tissue was evident at both time points.

Semi-quantitative assessment using the O’Driscoll scoring system (Table 2) confirmed these findings. Intact cartilage samples achieved the maximum score (24/24, Figure 2B). The empty defect group showed significantly lower scores at both 4 (median: 3.5/24) and 12 weeks (2/24) compared to the intact group (*p* < 0.05). The Col-PLA scaffold group had the highest scores among the surgical groups at both 4 weeks (7/24) and 12 weeks (8/24), with a significant improvement over the empty defect group at 12 weeks (*p* < 0.05). In the nanoenabled Col-PLA group, the median O’Driscoll score increased from 3/24 at 4 weeks to 6/24 at 12 weeks, indicating a trend toward improved cartilage repair over time. No statistically significant differences were observed between the Col-PLA and nanoenabled Col-PLA groups at either time point.

Picrosirius red staining observed under polarized light provided additional insights into the composition and organization of collagen fibers (Figure 2C). The empty defect group showed predominantly thick, disorganized orange fibers, consistent with collagen type I. In comparison, tissue in the Col-PLA and nanoenabled Col-PLA groups appeared more organized. Brightly birefringent residual scaffold fibers were visible in both groups, confirming partial persistence and integration into the surrounding tissue. Quantitative analysis of collagen composition revealed that defects treated with nanoenabled Col-PLA scaffolds had a significantly lower proportion of orange fibers at 4 weeks and a significantly higher proportion of green fibers at both 4 and 12 weeks compared to the empty defect group (*p* < 0.05, Figure 2D). These results suggest a reduced presence of collagen type I and an increased deposition of collagen type II, indicating more hyaline-like matrix characteristics in the nanoenabled Col-PLA-treated defects.

### 3.5 Synovial membrane and fluid

**FIGURE 3:**
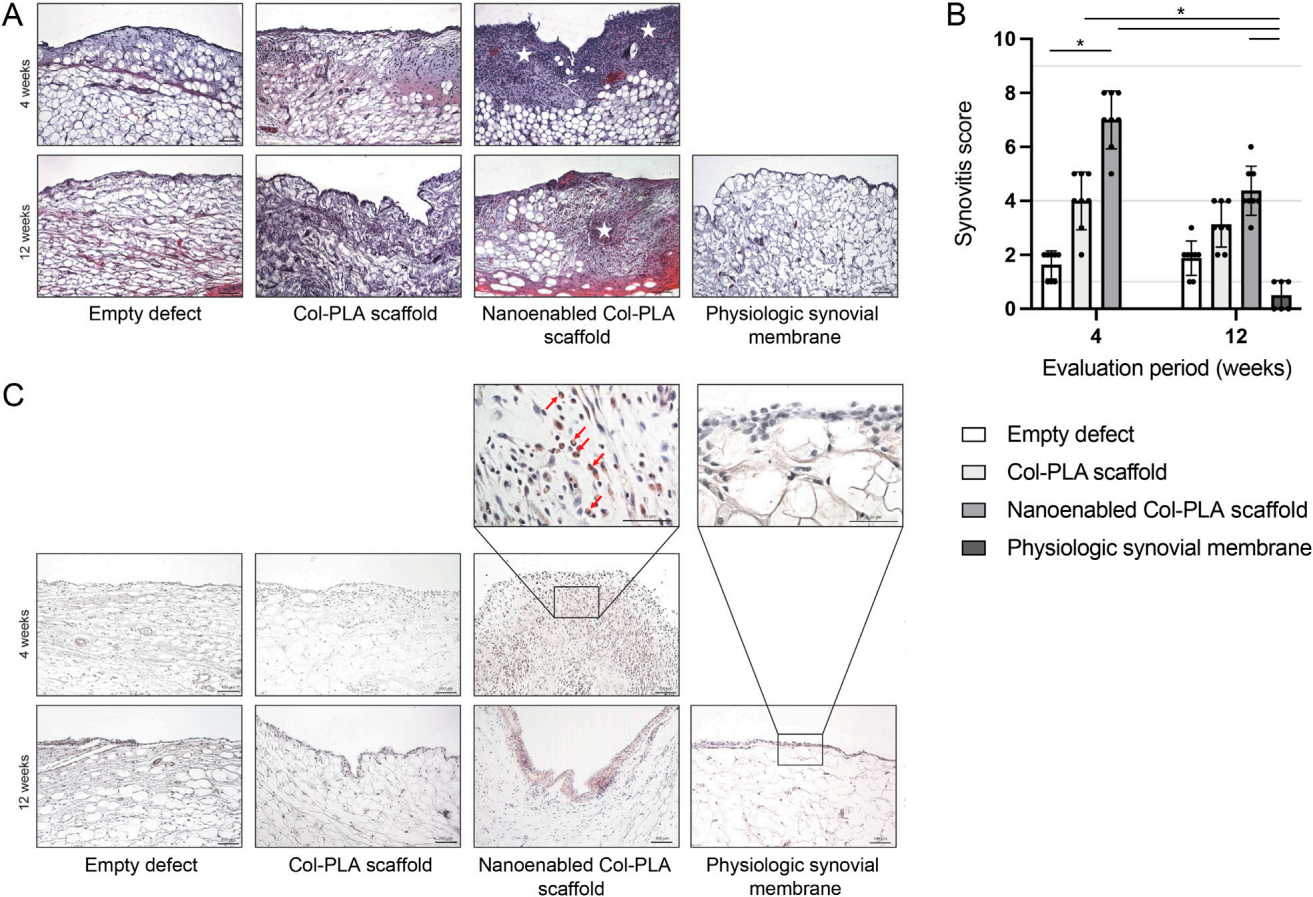
Histological assessment of synovial membrane inflammation. **(A)** Representative hematoxylin and eosin-stained sections of the synovial membrane from the empty defect, Col-PLA scaffold, and nanoenabled Col-PLA scaffold groups at 4- and 12-week post-surgery, alongside physiologic synovial membrane from non-operated animals. Stars indicate areas of immune cell infiltration. Scale bar: 100 µm. **(B)** Semi-quantitative evaluation of synovial inflammation using the Krenn synovitis score. Data are presented as median ± interquartile range (n = 6–8). Statistical analysis was performed using the Kruskal–Wallis test followed by Dunn’s multiple comparisons test; *p* ≤ 0.05. **(C)** Representative immunohistochemical images of CD8 staining for T lymphocytes in the synovial membrane. Scale bars: 100 μm and 50 µm.

Histological analysis of the synovial membrane in the non-operated (intact) group revealed a physiological architecture, characterized by normal cellularity and the absence of inflammation or synovitis (Figures 3A,B). Low-grade synovitis was observed in both the empty defect and Col-PLA scaffold groups at 4- and 12-week post-surgery (Figure 3B). At 4 weeks, the nanoenabled Col-PLA group showed a higher synovitis score than the Col-PLA group, although the difference was not statistically significant. Importantly, the synovitis score in the nanoenabled Col-PLA group decreased from high at 4 weeks to low levels by 12 weeks, comparable to the empty defect and Col-PLA groups, suggesting resolution of the initial inflammatory response. Notably, the nanoenabled Col-PLA group exhibited a significantly higher synovitis score compared to the empty defect group at 4 weeks (*p* < 0.05). Histological sections at this time point revealed synoviocyte hyperplasia and hypertrophy, accompanied by dense, band-like inflammatory cell infiltrates within the synovial membrane. Immunohistochemical staining further confirmed the presence of CD8^+^ T cells in the synovial tissue at both 4- and 12-week post-implantation (Figure 3C).

To evaluate the local inflammatory response associated with the different treatment conditions, levels of key pro-inflammatory cytokines and cartilage degradation markers were measured in synovial fluid at 4- and 12-week post-surgery. Overall, no marked differences were observed in the levels of IL-1β, PGE_2_, TNF-α, or MMP-13 among the surgical groups at either evaluation time point and all values remained within the physiological range observed in non-operated animals (Table 4). Only two statistically significant differences were observed. In the nanoenabled Col-PLA scaffold group, IL-1β concentrations were significantly lower at 4 weeks compared to the non-operated control group (8.6 ± 2.7 pg/mL vs. 28.0 ± 15.7 pg/mL, *p* < 0.05), but this difference was not observed at 12 weeks. In the empty defect group, MMP-13 levels significantly increased from 5.3 ± 1.5 pg/mL at 4 weeks to 11.9 ± 5.2 pg/mL at 12 weeks. However, all values remained within the physiological range observed in non-operated animals.

**TABLE 4:** Concentrations of cytokines and MMP-13 in synovial fluid from rabbits at 4- and 12-week post-surgery, and from non-operated control animals.

Cytokines /MMP-13	Plasma concentration in pg/mg						
	4 weeks after surgery			12 weeks after surgery			No surgery
	Empty defect	Col-PLA scaffold	Nanoenabled Col-PLA scaffold	Empty defect	Col-PLA scaffold	Nanoenabled Col-PLA scaffold	
IL-1β	14.6 ± 5.9	16.7 ± 5.1	8.6 ± 2.7^a^	17.9 ± 10.9	22.1 ± 8.6	19.8 ± 6.6	28.0 ± 15.7
PGE_2_	86.0 ± 69.4	223.8 ± 150.3	126.4 ± 70.8	250.2 ± 206.7	223.3 ± 104.4	137.2 ± 42.0	221.8 ± 135.6
TNF-α	9.0 ± 4.1	9.2 ± 2.8	7.3 ± 2.1	7.6 ± 3.3	5.9 ± 2.0	3.2 ± 0.5	7.6 ± 3.0
MMP-13	5.3 ± 1.5	6.6 ± 2.6	10.2 ± 6.5	11.9 ± 5.2^b^	6.0 ± 1.2	5.7 ± 2.7	10.8 ± 8.2

Inflammatory mediators in the synovial fluid of rabbits with empty osteochondral defects and osteochondral defects treated with Col-PLA scaffolds or nanoenabled Col-PLA scaffolds after 4 and 12 weeks as well as untreated rabbits that did not undergo surgery. Data are presented as the means ± SD. *n* = 3–8. IL-1β, interleukin-1 beta; PGE_2_, prostaglandin E2; TNF-α, tumor necrosis factor-α; MMP-13, matrix metalloproteinase-13.

^a^ *p* < 0.05 (compared to the intact cartilage group at 4 weeks); ^b^ *p* < 0.05 (compared to the empty defect group at 4 weeks).

## 4 Discussion

The implantation of tissue-engineered constructs to repair cartilage defects after traumatic injury carries the risk of surgical site infection and may provoke an inflammatory response that can contribute to further cartilage degradation (Wei et al., 2021). Clinical procedures for degenerative cartilage conditions, such as ACI, are currently limited by a lack of strategies to modulate the inflammatory microenvironment. In this context, immunomodulatory approaches, such as integrating nanomaterials into three-dimensional scaffolds for local drug delivery, may offer more effective therapeutic options (Leite Pereira et al., 2022). In a previous study, a Col-PLA scaffold functionalized with ibuprofen-loaded nanoparticles showed anti-inflammatory effects both *in vitro* and in a mouse air pouch model (Pereira Vasconcelos et al., 2024). The aim of the present study was to evaluate the regenerative capacity of an advanced nanoenabled Col-PLA scaffold, loaded with a combinatorial therapeutic payload, ibuprofen (anti-inflammatory), BB-94 (anti-proteolytic), and mupirocin (anti-bacterial), following implantation into osteochondral defects in rabbits.

Across all surgical groups, macroscopic and histological analyses revealed signs of tissue regeneration. Empty defects exhibited poor healing with a tendency to deteriorate over time, whereas Col-PLA and nanoenabled Col-PLA scaffold-treated defects showed progressive regeneration between weeks 4 and 12, as observed in macroscopical and histological evaluation. These time points are commonly used in preclinical cartilage repair studies to assess both early host response and the progression of matrix regeneration and scaffold degradation (Hunziker, 2002; Mainil-Varlet et al., 2003; Wang et al., 2015). The highly porous architecture of both Col-PLA and nanoenabled Col-PLA scaffolds appeared to facilitate cell adhesion and infiltration, and residual scaffold fibers were well integrated with host tissue at both evaluation points. Such integration is crucial for maintaining implant functionality (Yari et al., 2022), and contrasts with reports of inadequate tissue integration or poor degradation seen with other biomaterials (Hutmacher, 2000; Abarrategi et al., 2010). The observed homogeneous host cell distribution within the scaffolds may offer advantages over traditional two-stage, cell-based strategies such as ACI by Brittberg et al. (1994), enabling a one-step approach that reduces patient risk and healthcare costs.

Although the biomechanical properties of regenerated tissue did not reach those of intact hyaline cartilage, this was expected. These scaffolds were not intended to replicate native cartilage mechanics but rather to provide a conducive environment for cell migration, ECM production, and eventual scaffold resorption. The biomechanical data support the notion that both scaffold types facilitated regenerative tissue formation better than untreated controls. Contrary to the initial hypothesis, the nanoenabled Col-PLA scaffold did not significantly outperform the Col-PLA scaffold in terms of cartilage regeneration. However, a transient inflammatory response was observed in the synovial membrane of the nanoenabled group at 4 weeks, characterized by higher synovitis scores and CD8^+^ T cell infiltration. This response resolved by 12 weeks, aligning with prior knowledge that biomaterial implantation naturally elicits an immune reaction, often moderated by scaffold composition and structure (Medzhitov, 2008; Salthouse et al., 2023). The likely source of this early immune response was the nanoemulsion-based drug delivery system used to functionalize the scaffold, as it was not observed in the Col-PLA-only group. A previous study using PLGA nanoparticles to deliver ibuprofen demonstrated anti-inflammatory efficacy in a mouse model (Pereira Vasconcelos et al., 2024); however, direct comparison with the current study is limited due to differences in drug carriers (PLGA vs. nanoemulsion), animal models, and time points. The presence of oil in the nanoemulsion, absent in PLGA nanoparticles, may have contributed to the observed inflammatory effects. Importantly, this transient synovial inflammation did not negatively impact overall osteochondral regeneration, as supported by macroscopic, histological, and mechanical assessments.

Additionally, synovial fluid analysis revealed no marked differences in pro-inflammatory cytokines among surgical groups, and levels remained within physiological ranges. IL-1β concentrations were significantly lower in the nanoenabled scaffold group compared to non-operated controls at 4 weeks, possibly reflecting early ibuprofen release. While ibuprofen is known to suppress IL-1β-induced PGE_2_ and nitric oxide production in chondrocytes (Li et al., 2019), the levels of PGE_2_ and TNF-α in this study remained within physiological ranges. This suggests that longer implantation periods may be necessary to fully distinguish the regenerative and immunomodulatory effects of nanoenabled scaffolds. The absence of significant changes in inflammatory markers may be attributed to the use of a healthy osteochondral defect model. In an osteoarthritic or inflammatory context, characterized by elevated levels of IL-1β, TNF-α, MMPs, and other catabolic mediators (Reinholz et al., 2004; Chow and Chin, 2020; Knights et al., 2023), the nanoenabled scaffold’s drug delivery system might exert more pronounced therapeutic effects.

Despite the incorporation of BB-94, a broad-spectrum MMP inhibitor, no anti-proteolytic effect on cartilage regeneration was observed in this healthy rabbit model. The absence of chronic inflammation likely limited the activation of matrix metalloproteinases, thereby reducing the therapeutic relevance of BB-94 in this context. Nonetheless, its inclusion may prove critical in future studies using OA or inflammatory models, where MMP-mediated matrix degradation is more pronounced. Similarly, although mupirocin was integrated for its anti-bacterial activity, no infection-related endpoints were assessed. Testing the scaffold in infection models could help further validate its potential, particularly given the clinical importance of implant-associated infections (Voss et al., 2021).

Recent advances in tissue-engineered cartilage repair have increasingly focused on multifunctional scaffolds incorporating bioactive molecules or nanomedicine-based strategies to modulate the local microenvironment. For example, Pang et al. (2025) engineered a cytokine-activated, mesenchymal stem cell-derived ECM scaffold that was shown to enhance chondrogenesis and support hyaline cartilage regeneration via intrinsic growth factor and cell-recruiting cues. Xiang et al. (2025) explored tryptophan-derived small molecules that modulate serotonergic pathways to stimulate matrix production and cartilage homeostasis. In a complementary direction, a study by Wang Y et al. (2024) investigated a hydrogel scaffold capable of absorbing endogenous bone morphogenic protein-2, promoting matrix deposition in a rat osteochondral defect model. While these approaches have demonstrated promising outcomes via biological or metabolic modulation, the present study is distinguished by the application of a tricombinatorial drug-loaded, nanoemulsion-based Col-PLA scaffold that concurrently delivers anti-inflammatory (ibuprofen), anti-proteolytic (BB-94), and anti-bacterial (mupirocin) agents. This therapeutic combination was selected to address three clinically critical barriers to successful cartilage repair: inflammation, matrix degradation, and infection. Importantly, unlike single-agent systems, this novel scaffold offers simultaneous and localized delivery of multiple mechanistically distinct agents, enhancing its potential to stabilize the microenvironment and support long-term regeneration. Compared to other studies that utilize either endogenous signaling cues or passive bioactive coatings, the present approach introduces a versatile, modular platform that can be customized for diverse pathological contexts.

A limitation of this study is that no long-term release kinetics studies of the drugs incorporated into the nano-enabled Col-PLA scaffold were performed. We anticipate that drug release from the nanoenabled scaffolds will occur in a gradual and sustained manner over time, simultaneously with the degradation of the composite. However, further release experiments are required before advancing to an *in vivo* inflammation model.

In summary, both the Col-PLA and nanoenabled Col-PLA scaffolds supported earlier osteochondral repair compared to untreated defects. While no additional regenerative benefit was observed for the nanoenabled variant under physiological conditions, its immunomodulatory and anti-microbial design offers promising potential for more complex clinical scenarios. Further evaluations in disease-relevant and long-term models are warranted to fully realize the therapeutic applicability of nanoenabled Col-PLA scaffolds.

## 5 Conclusion

This study demonstrated the regenerative potential of a novel nanoenabled Col-PLA scaffold for osteochondral defect repair following *in vivo* implantation in a rabbit model. Both the Col-PLA and nanoenabled Col-PLA scaffolds supported host cell adhesion, infiltration, and tissue regeneration, exhibiting superior performance compared to untreated defects. Although the nanoenabled Col-PLA scaffold did not show a significant advantage in cartilage regeneration over its non-functionalized counterpart in this model, its immunomodulatory, anti-proteolytic and anti-bacterial equipment may offer added benefits in more complex or clinically relevant scenarios. Future investigations in osteoarthritic or infection models are warranted to further validate the efficacy of the nanoenabled Col-PLA scaffold and advance its translational potential in regenerative medicine.

## Data Availability

The raw data supporting the conclusions of this article will be made available by the authors, upon reasonable request.
