# Supplementary material for: Regenerative potential of nanoenabled collagen-polylactide scaffolds for osteochondral defect repair in rabbits
Source: Front Bioeng Biotechnol. 2025 Dec 1;13:1699338. doi: 10.3389/fbioe.2025.1699338 (PMC12702961; doi:10.3389/fbioe.2025.1699338)
Supplement: Supplementary file 1 [file DataSheet1.pdf]

Supplementary Material

1 Supplementary Materials and Methods

1.1 Synthesis and characterization of methacrylate-functionalized dextran polymer (DXT-MA)

1.1.1 Preparation of methacrylate-functionalized dextran polymer (DXT-MA)

As previously described, to produce dextran single chain polymer nanoparticles, methacrylate-functionalized dextran polysaccharide (DXT-MA) with a degree of substitution of methacrylate groups (DS_{DXT-MA}, percentage of modified hydroxyl groups per repeating unit) of 40% were produced. First, dextran (1g, DXT-40, Pharmacosmos, pharmaceutical grade) was dissolved in 30 mL of dimethyl sulfoxide (DMSO) under a nitrogen atmosphere. To this solution, 200 mg of 4-(*N*,*N*-dimethylamino)pyridine (DMAP, 1.6 mmol, Acros-Organics) was added. Then, 1 mL of glycidyl methacrylate (GMA, 1.2 mmol, Aldrich) was incorporated and the mixture was stirred at room temperature. After 4 days, the reaction mixture was quenched with 0.1M HCl and purified by dialysis against ultrapure water (MWCO 3,500 Da) at room temperature until deionized water conductivity value of < 1µS was reached (9 days, refreshing with 4 L of deionized water twice per day).

1.1.2 Preparation of dextran-based single-chain polymer nanoparticles (DXT-SCPN-MA)

For the intra-crosslinking reaction to prepare dextran-based single chain polymer nanoparticles (DXT-SCPN-MA, Figure S1), 0.413 mL of a previously prepared 0.15 M solution (2 mL, MeOH/PBS, 1:1, v/v, adjusted to pH= 9.5) of crosslinker 2,2'-(ethylenedioxy)diethanethiol (DODT, 0.06 mmol, 10.1 µL, Aldrich) was added dropwise using a syringe pump (0.04 mL/h) to a 0.02 M solution of DXT-MA (DS_{DXT-MA}=40%, 100 mg, 0.025 mmol of MA, 13 mL PBS, adjusted to pH= 9.5) during 8 h at room temperature and under constant stirring. After addition, the reaction was maintained under stirring at room temperature for 12 h. A 5 mL fraction was purified by dialysis against ultrapure water (MWCO 3500 Da) until deionized water conductivity values of < 1µS were achieved (5 days, refreshing with 4 L of deionized water twice per day). Finally, the resulting aqueous solution was freeze-dried to obtain nanoparticles as a white solid. Yield >90%. ^1H NMR spectrum (Figure S2, 500 MHz, D_2O; δ ppm): 6.34-6.12 (m, 1H, methacrylic-CH), 5.94-5.70 (m, 1H, methacrylic-CH), 5.55-4.85 (4.31H, including H-1 and H-2/3 MA-substituted), 4.34-3.28 (28.29H, m, rest of Glc and $2xCH_2O$ of cross-linker), 3.06-2.53 (4.97H, m, $CH(CH_3)CH_2S$, CH_2S of cross-linker), 3 (s, 3H, methacrylic-CH_3), 3.1(s, 3H, cross-linker-CH_3). Dh (DLS) = 13 ± 2 nm; PDI 0.24 (Figure S2).

1.1.3 Determination of the degree of substitution of methacrylate group of the DXT-SCPN-MA ($DS_{SCPN-MA}$)

For the calculation the degree of substitution of the methacrylate group of the DXT-SCPN-MA, the most representative signals for DS calculation in the ^1H NMR (500 MHz, D_2O) spectrum are those at 6.35-6.10 ppm (m, 1H, TRANS-methacrylic-CH), 5.92-5.72 ppm (m, 1H, CIS-methacrylic-CH) and 4.20-3.33 ppm (28.29 H, m, rest of hydrogen atoms of Glc (Figure S2). Reference signals for both molecules involved in the reaction are required. DS calculation requires the subtraction of 5H corresponding to the substituted Glc in the region of 3.3-4.2 ppm (28.29 – 5 = 23.29). This difference

corresponds to 6 Hydrogen atoms of unmodified Glc. Integration of one single proton present in unmodified Glc moiety was obtained by the subsequent division against the total number of protons involved in the integral (23.29 / 6 = 3.88). DS was calculated as the ratio between the substituted Glc and the sum of both integrations (Glc + $Glc_{substituted}$) following the formula:

Equation 1. DS calculation example:

$$DS(\%) = \frac{Glc_{substituted}}{Glc + Glc_{substituted}}\, x\, 100 = \frac{1}{3.88+1}\, x\, 100 = 20\ \%$$

In summary the DXT-SCPN-MA, the final $DS_{SCPN\text{-}MA}$ was calculated to be 20%, showing that 50% of the DXT-MA were used for the intra-crosslinking reaction to form the single polymer nanoparticles.

1.2 Microcomputed tomography

Following biomechanical testing, the femoral condyles were fixed in 4% saline-buffered formalin solution and microcomputed tomography (µCT) was performed (Skyscan 1172, Skyscan, Kontich, Belgium) at a resolution of 17 µm and a voltage of 100 kV and 100 µA. With each specimen, two phantoms with a defined density of hydroxyapatite (250 mg/cm^3 and 750 mg/cm^3) were scanned to convert the attenuation coefficients of the voxels into bone mineral density. Two-dimensional and three-dimensional (3D) images were obtained using Skyscan software (NRecon version 1.7.1.0, CTvox version 3.3.0). The subchondral bone was qualitatively assessed for osteolysis and/or subchondral bone cyst formation.

1.3 Biomechanical scaffold characterization

The compressive properties of collagen-polylactide (Col-PLA) and nanoenabled Col-PLA scaffolds were assessed as previously described (Saranya et al., 2023). Briefly, a material testing machine (Z10, ZwickRoell, Germany) was used for the biomechanical tests (Figure 1 and Figure S4). Two cylindrical samples (Ø 4.8 mm) were punched out of the scaffolds using a biopsy punch (Stiefel Laboratories Inc., UK) and placed one on top of the other in a phosphate buffer saline (PBS)-filled test chamber which provided confined conditions. A porous ceramic (Al_2O_3) cylinder was positioned above the samples to allow uniaxial fluid flow through the 3D matrix during testing. A stainless-steel punch applied a defined compressive strain rate, while a 20 N load cell (ZwickRoell) recorded the resulting force. A preload of 0.1 N ensured consistent initial conditions, and the combined sample thickness (h_0) was automatically measured. To account for viscoelastic behavior under different compression levels, samples were tested sequentially at three strain rates (ε = 0.1, 0.15, 0.2) using a loading rate of 3% $h_0$min, which reflected physiological (10–15%) and pathological (20%) conditions (Wong et al., 2008; Lai and Levenston, 2010). Each load was maintained for 60 minutes to allow relaxation. Immediately after the last strain rate was completed, cyclic loading was applied to mimic dynamic conditions with 10 cycles of sinusoidal compression at ε_{max} = 0.25 and 1 Hz (gait frequency). Two key viscoelastic parameters were extracted from the multi-step relaxation tests using MATLAB R2020a (Mathworks, USA). The equilibrium modulus (E_{eq}) was calculated as the ratio of equilibrium stress to applied strain (Equation 2):

Equation 2:

$$E_{eq} = \frac{\sigma_{t\to\infty}}{\varepsilon_i};\ \varepsilon_i = 0.1; 0.15; 0.2$$

The hydraulic permeability (k), indicating resistance to fluid flow, was obtained by fitting Mow's diffusion equation using non-linear least squares at all three strain levels (Mow and Huiskes, 2005) (Equation 3):

Equation 3:

$$\sigma_t = \sigma_{t\to\infty} + 2 \cdot H \cdot \varepsilon_i \cdot e^{\left(-\left(\frac{\pi}{h_0}\right)^2 \cdot H \cdot k \cdot t\right)}$$

From cyclic loading, the storage modulus (E′) and loss modulus (E″) were determined. E′ reflects elastic behavior, while E ″ indicates viscous behavior. The phase difference (δ) between stress and strain was used to derive these values which were averaged over 10 cycles. (Reuter and Ponomarev, 2014).

Equation 4:

$$E' = \frac{\sigma_0}{\varepsilon_0} cos\delta$$

Equation 5:

$$E'' = \frac{\sigma_0}{\varepsilon_0} sin\delta = E' \cdot tan\delta$$

Differences between the Col-PLA and nanoenabled Col-PLA were analyzed using Mann-Whitney U testing at a significance level of $p < 0.05$.

1.4 High-performance liquid chromatography (HPLC) analyses

High-performance liquid chromatography (HPLC) analyses were performed using an Agilent 1100 Series (G1316A) instrument to quantify drug loading. Samples (10 µL) were injected onto a ZORBAX Eclipse XDB-C18 column (4.6 × 150 mm) and separated using a gradient elution with Mobile Phase A (Milli-Q® water containing 0.1% H_3PO_4) and Mobile Phase B (acetonitrile containing 0.1% H_3PO_4). The gradient program was as follows: 0–3.5 min, 90% A and 10% B; 3.5–9 min, 5% A and 95% B; 9–12 min, 90% A and 10% B. The flow rate was 0.6 mL/min, and the total run time was 12 min. UV detection was performed at 210 nm (ibuprofen at 7.4 min and Mupirocin 7.9 min) and 254 nm (BB-94) at 7.4 min). All samples were filtered through a 0.22 µm nylon membrane prior to injection.

1.5 Release studies of drugs from loaded nanoemulsions

To study drug release, a loaded nanoemulsion was immersed in a 2 mL Eppendorf tube containing 2 mL of deionized water as the receptor medium. The tube was placed in a roller mixer under gentle agitation, and every hour 1.5 mL of the receptor solvent was withdrawn and replaced with fresh deionized water. Drug loading was quantified by HPLC. For ibuprofen, the samples were directly injected into the HPLC system. For mupirocin and BB-94, the collected samples were freeze-dried and redispersed in DMSO prior to injection. Quantification was performed using previously prepared calibration curves for each drug. The drug release profiles from the scaffolds are shown in Figure S4.

1.6 Scanning Electron Microscopy analysis of the nanoenabled material

Scanning electron microscopy (SEM) examination was performed using a high resolution (Schottky) environmental scanning electron microscope with X ray microanalysis and electron backscattered diffraction analysis (FEI Quanta 400 FEG SEM/EDAX Genesis X4M). Samples were coated with and Au/Pd thin film by sputtering using the SPI Module Sputter Coater equipment. Images were obtained at magnification of 100×, 500× and 10000×.

2 Supplementary Results

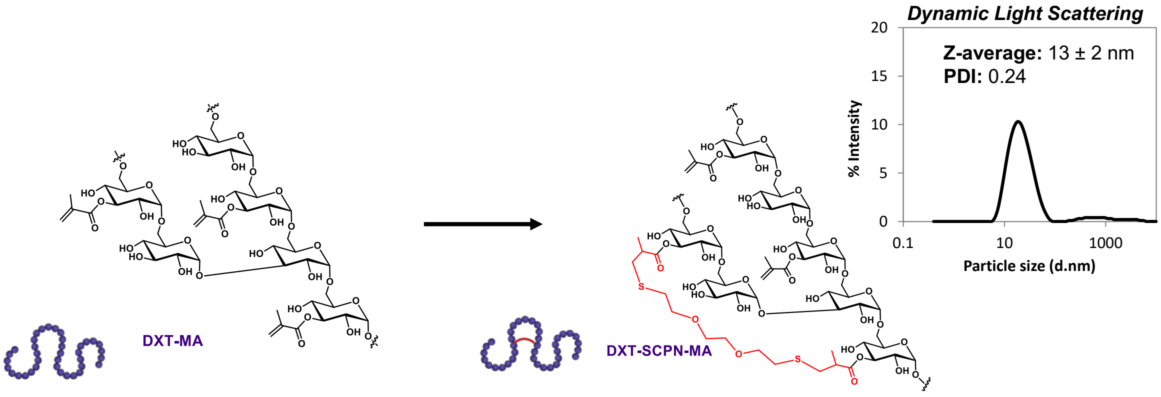

Supplementary Figure S1. Reaction scheme for the synthesis of methacrylated dextran single chain polymeric nanoparticles (DXT-SCPN-MA) from DXT-MA with DS_{MA} of 40% dissolved in PBS adjusted at pH 9.5 at 0.02 M by dropwise addition of 2 mL, MeOH/PBS, 1:1, v/v, adjusted to pH= 9.5 of crosslinker 2,2’-(ethylenedioxy)diethanethiol (DODT, 0.06 mmol, 10.1 µL, Aldrich). The particles diameter was measured by dynamic light scattering.

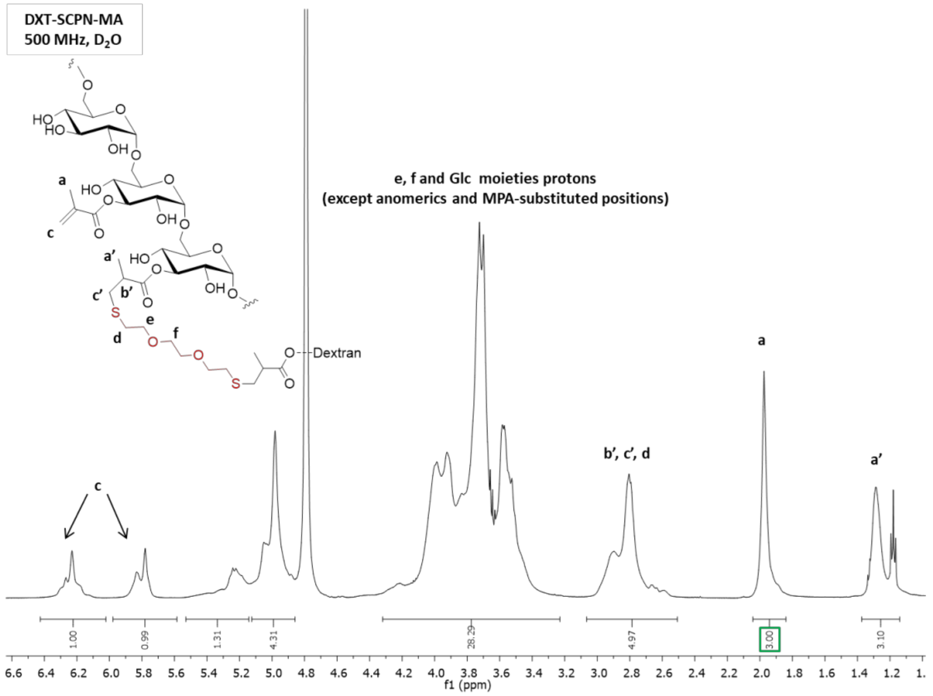

Supplementary Figure S2. ^{1}H NMR spectrum (D_2O, 500 MHz) of DXT-SCPN-MA and the assignation of each signal to the proton of DXT-SCPN-MA. Note the signal at 2 ppm of the methyl group of the methacrylate group taken as reference (3 protons) for DS calculation (Equation 1).

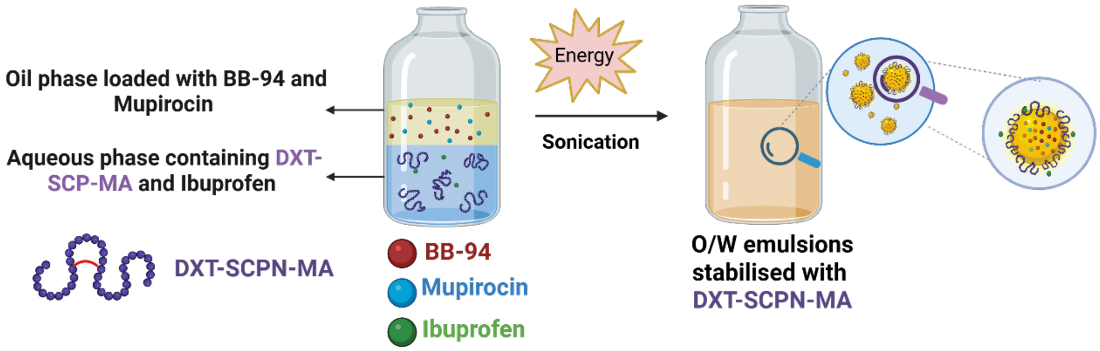

Supplementary Figure S3. Schematic representation of the production via sonication of oil-in-water (O/W) nanoemulsions stabilised by DXT-SCPN-MA and loaded with BB-94 and mupirocin, in the presence of ibuprofen in the aqueous phase.

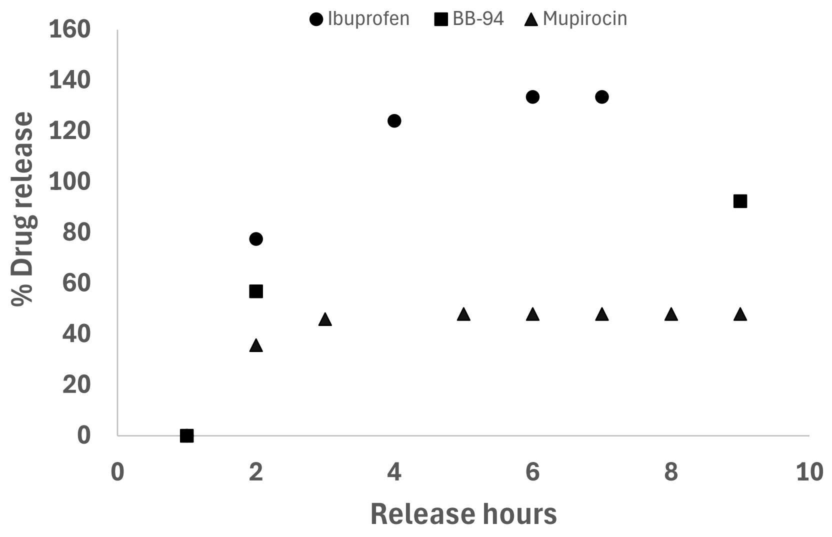

Supplementary Figure S4. In vitro release studies of loaded nanoemulsions in water at 37°C. The graph shows the cumulative drug release over time. Quantification of the drugs was determined by high performance liquid chromatography. ●) Ibuprofen release from tricombinatorial nanoemulsion (Ibuprofen/BB-94/Mupirocin drugs combination). ■) BB-94 release from tricombinatorial nanoemulsion. ▲) Mupirocin release from tricombinatorial nanoemulsion.

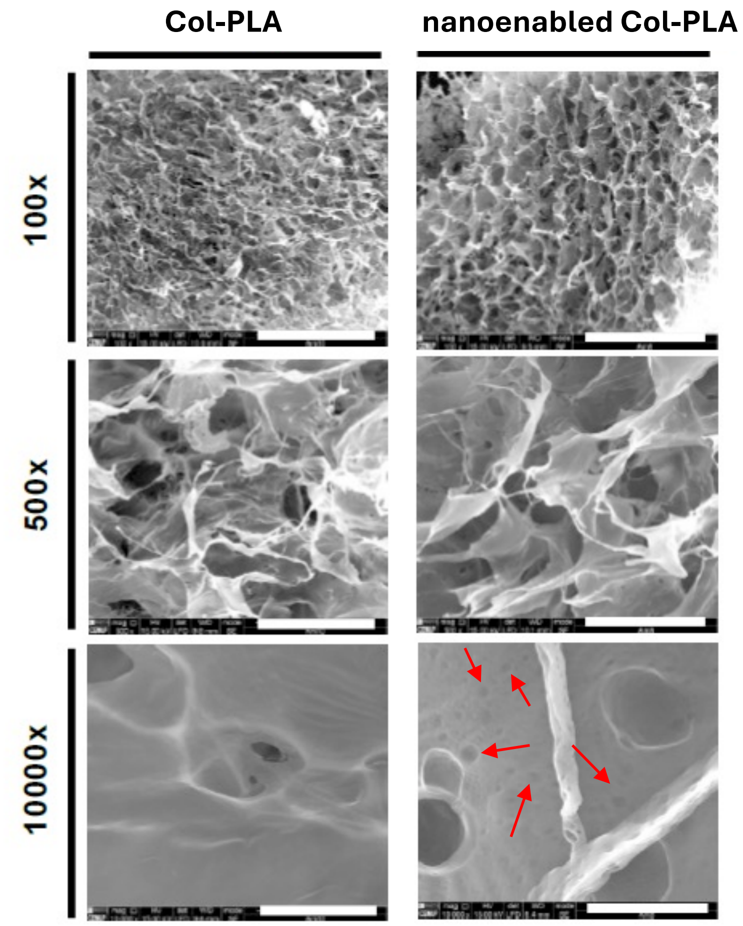

Supplementary Figure S5. Scanning electron microscopy (SEM) images at 100×, 500× and 10000× magnification for plain collagen scaffold (Col-PLA) and nanoenabled scaffold with emulsions. Scale bars: 100× – 1 mm; 500× – 200 µm; 10000× – 10 µm are represented in each image. The presence of emulsions in the SEM images is clearly observed at maximum magnification (10000×), primarily in the upper left area of the image. Some of the nanoemulsions shown in the image have been indicated with red arrows.

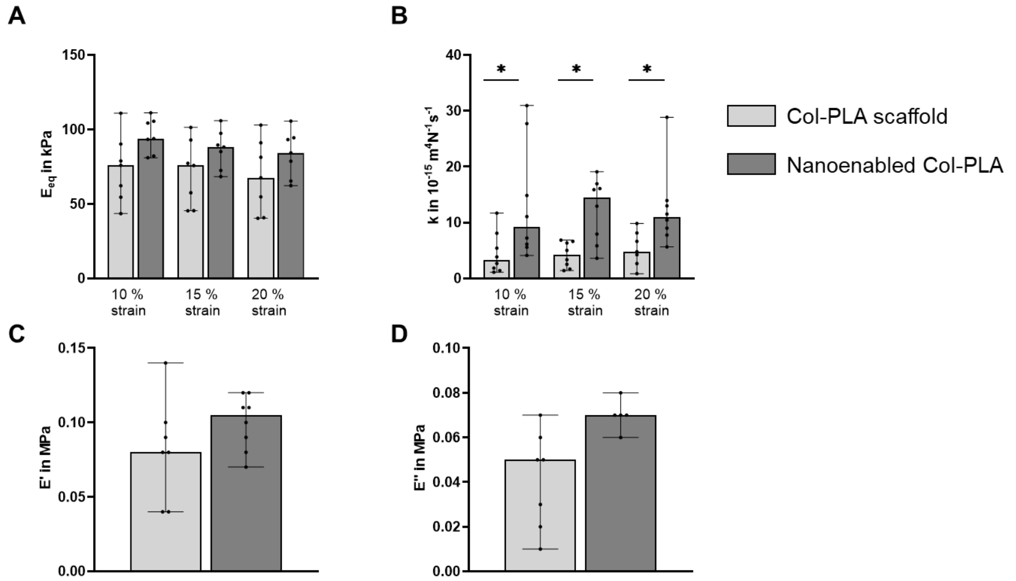

Supplementary Figure S6. Biomechanical properties of Col-PLA and nanoenabled Col-PLA scaffolds. **(A)** Equilibrium modulus (E_{eq}) and **(B)** permeability (k) were assessed at 10%, 15%, and 20% strain. Data points for **(C)** loss modulus (E′) and **(D)** storage modulus (E″) represent the mean values across n = 10 applied cycles. Non-parametric statistical analysis was performed (n = 7–8). $*p < 0.05$.

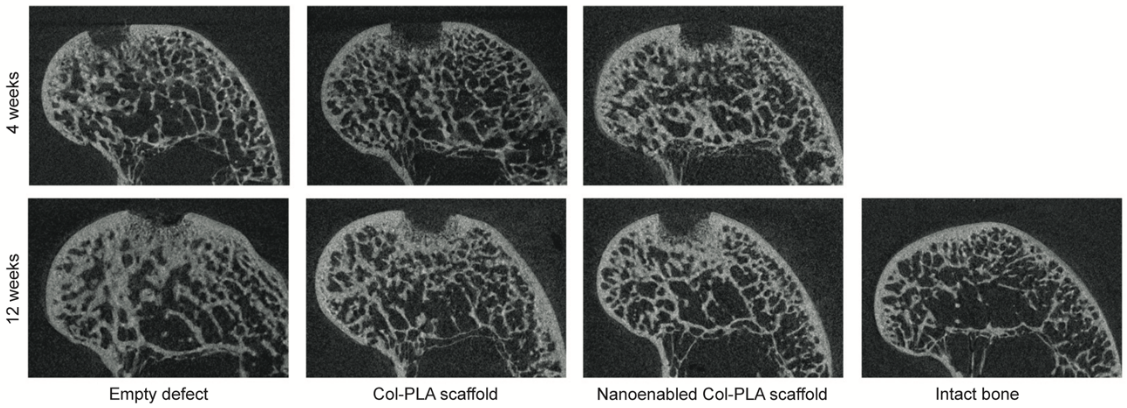

Supplementary Figure S7. Representative two-dimensional µCT images of the medial femoral condyle are shown for osteochondral defects left untreated (empty), treated with either the Col-PLA scaffold or the nanoenabled Col-PLA scaffold at 4- and 12-weeks post-surgery, as well as for intact (non-operated) bone. All groups exhibit signs of subchondral bone regeneration without evidence of cyst formation. Scale bar: 5 mm.
